# Supplementary material for: Genetic map of regional sulcal morphology in the human brain from UK biobank data
Source: Nat Commun. 2022 Oct 14;13:6071. doi: 10.1038/s41467-022-33829-1 (PMC9568560; doi:10.1038/s41467-022-33829-1)
Supplement: Supplementary file 1 — Supplementary Information [file 41467_2022_33829_MOESM1_ESM.pdf]

Supplementary Information

Table of Contents

List of supplementary files 2
Supplementary Figures 3
Summary of the reliability of the sulcal measurements 13
Empirical estimation of false discoveries 14
Sensitivity analyses 16
Missense variant in SLC6A20 transporter highlights role of glycine and proline pathways in brain sulcal width modulation 18
Supplementary Table 1. Summary of brain imaging related studies in GWAS Catalog 20
Multi-trati colocalization (HyPrColoc) sensitivity analysis 21
Supplementary Table 2. Summary of neuropsychiatric and cognitive phenotypes tested for genetic correlation 22
Supplementary Table 3. Summary of prior peer-reviewed applications of BrainVISA to investigate brain sulcal morphology in human health and disease. 23
Biogen Biobank Team contributors 24
Supplementary References 25

Supplementary Figures

Supplementary Figure 1. t-SNE representations of brain sulci.

Supplementary Figure 2. GWAS Study overview.

Supplementary Figure 3. Effect size vs minor allele frequency (MAF) of sentinel associations across sides and shape parameters.

Supplementary Figure 4. SNP-based heritability estimates.

Supplementary Figure 5. Comparison of Z-scores between sides.

Supplementary Figure 6. Genetic and phenotypic correlation heatmap of local sulcal measures.

Supplementary Figure 7. Heatmap of gene expression across brain developmental stages.

Supplementary Figure 8. (a) Differential expression (GTEx v8 gene expression data) of the closest genes to each lead GWAS variant across adult tissues. Tissues with significant (FDR $p \leq 0.05$) up- and down- regulation of genes within the closest gene set compared with other tissues are shown in red. **(b) Differential expression (Zhong et al 2018 single-cell RNA-seq expression data) of the closest genes to each lead GWAS variant across early to mid-gestation fetal brain cells sampled between gestation weeks 8-26**. Cells with significant (FDR $p \leq 0.05$) enrichment of gene expression of the closest gene set compared with other tissues are shown in red.

Supplementary Figure 9. Colocalization heatmap of brain tissue *cis* eQTLs against significant brain sulcal associations.

Supplementary Figure 1. (a) t-SNE representation of brain sulci folds coloured by shape parameters. (b) t-SNE representation of sulcal measures in context of existing UKB brain imaging measures.

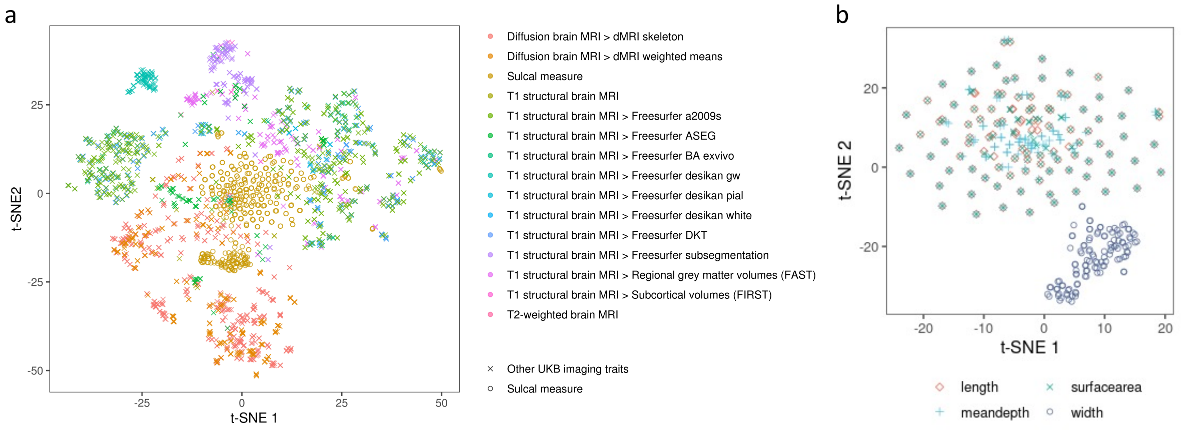

Supplementary Figure 2. GWAS Study overview.

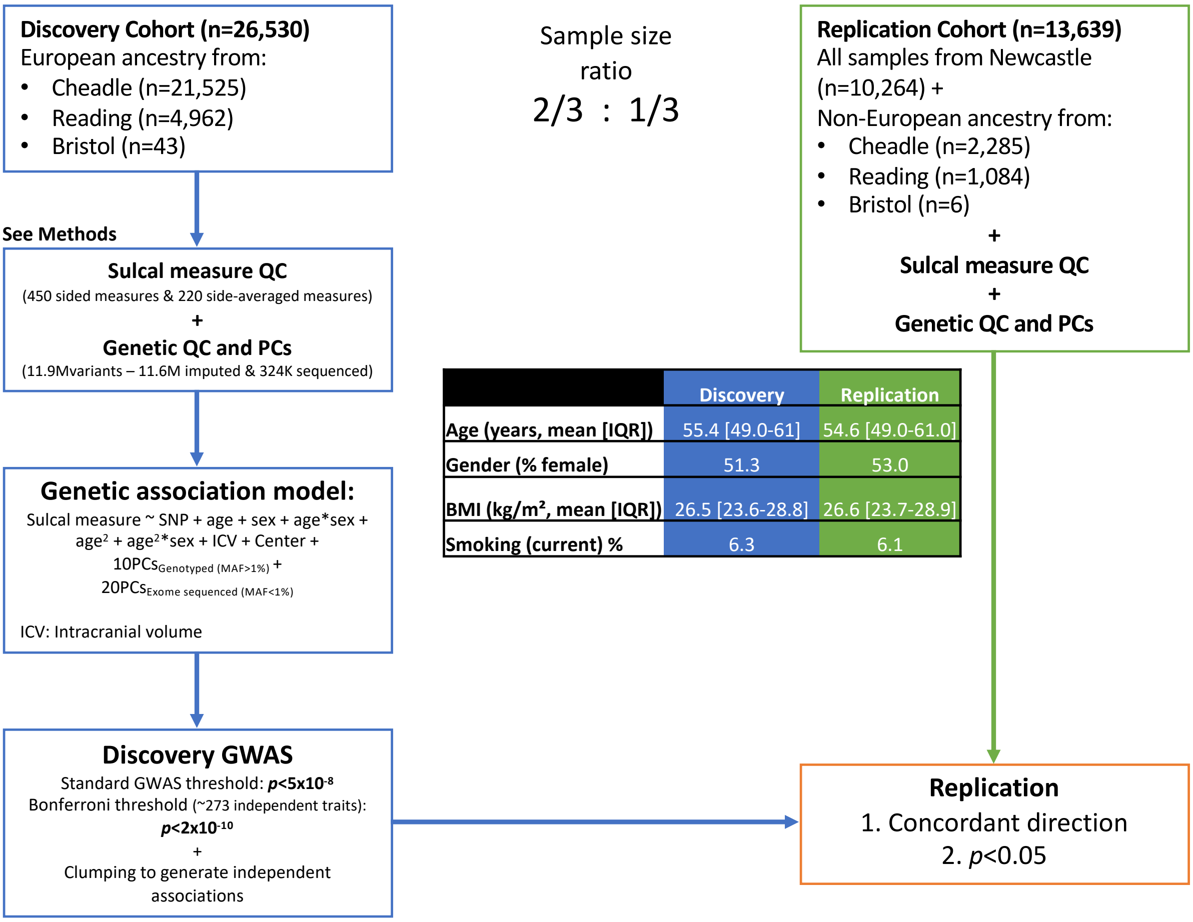

	Discovery	Replication
Age (years, mean [IQR])	55.4 [49.0-61]	54.6 [49.0-61.0]
Gender (% female)	51.3	53.0
BMI (kg/m², mean [IQR])	26.5 [23.6-28.8]	26.6 [23.7-28.9]
Smoking (current) %	6.3	6.1

Supplementary Figure 3. Effect size vs minor allele frequency (MAF) of sentinel associations across sides and shape parameters.

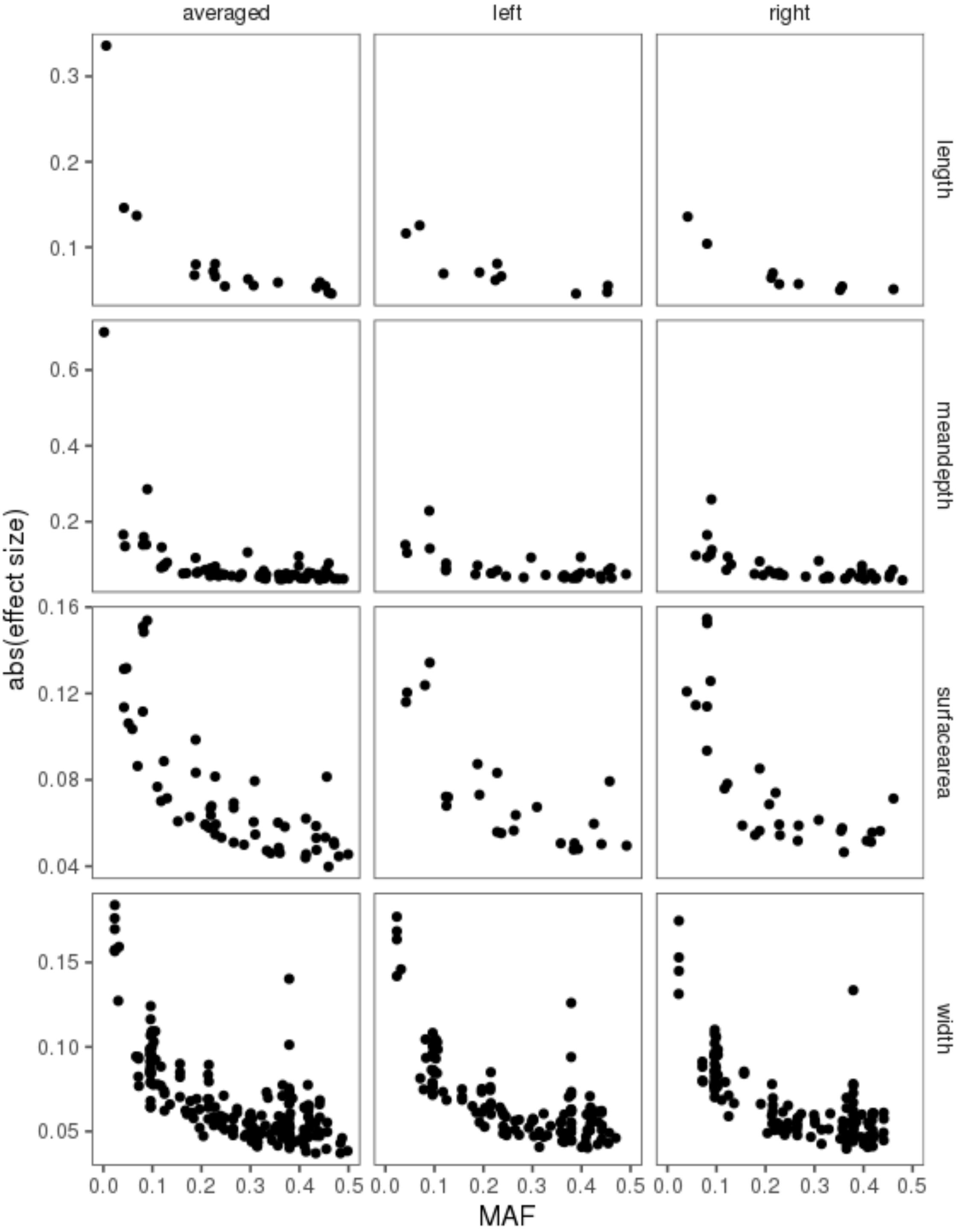

Supplementary Figure 4. SNP-based heritability estimates. Number of sulcal traits listed beneath the boxplot. Each box plot presents the median, first and third quartiles, with upper and lower whiskers representing 1.5x inter-quartile range above and below the third and first quartiles respectively.

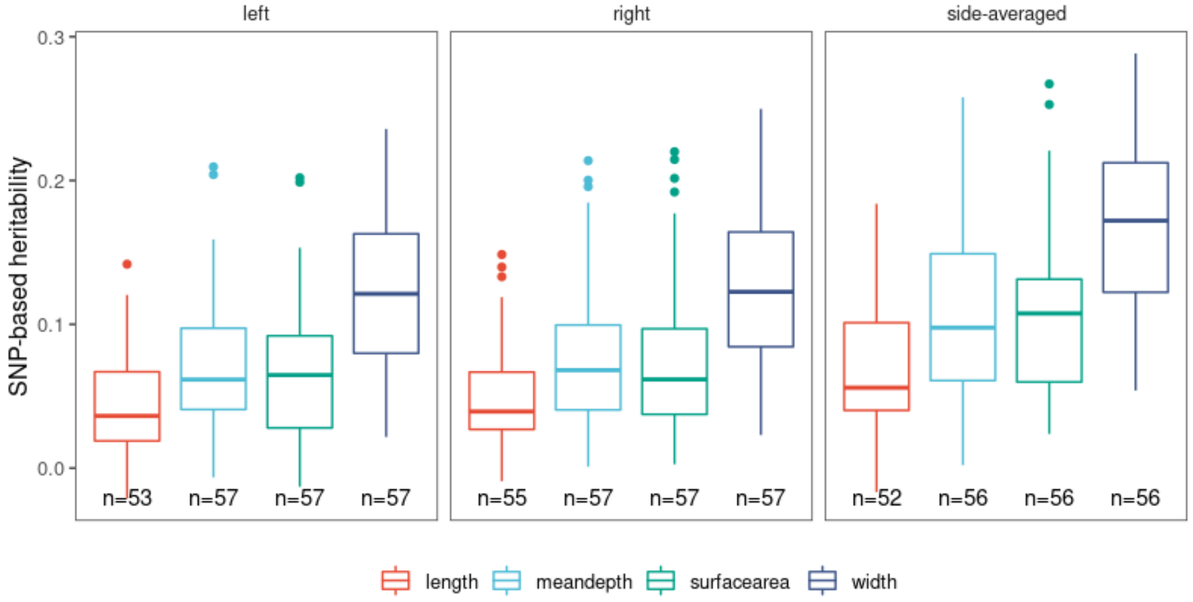

Supplementary Figure 5. Comparison of Z-scores between sides. Z_left, Z_right, Z_mean correspond to Z-scores of left, right and bilateral sulcal measures respectively.

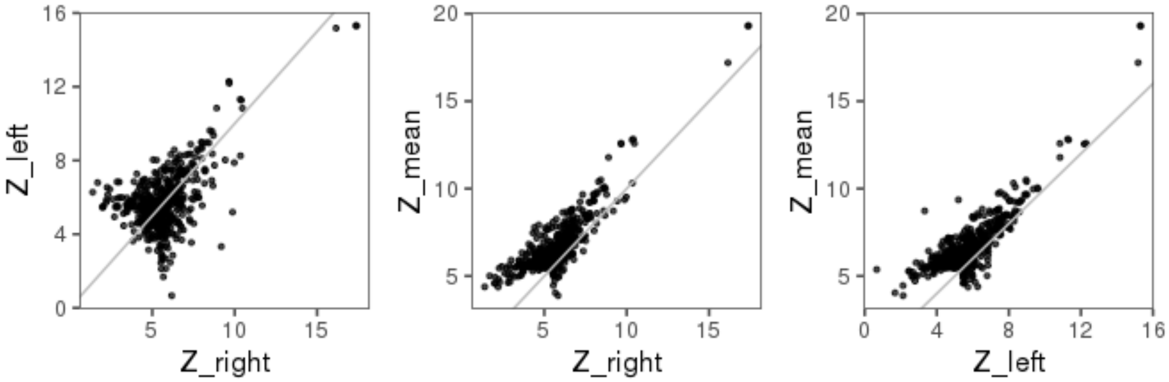

Supplementary Figure 6. Genetic (top triangle) **and phenotypic** (bottom triangle) **correlation heatmap of local sulcal measures.** Phenotypic correlations were calculated using Pearson's correlation.

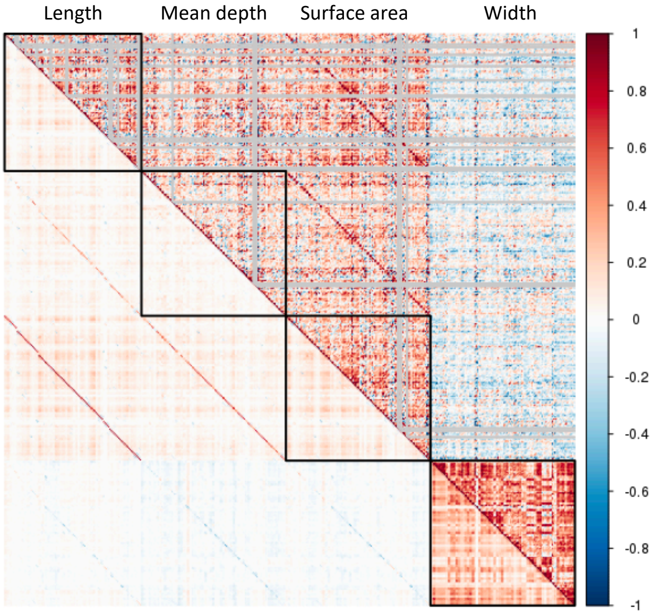

Supplementary Figure 7. Heatmap of gene expression across brain developmental stages. Log2 transformed BrainSpan expression values (RPKM; Read Per Kilobase per Million) for all genes in significant loci.

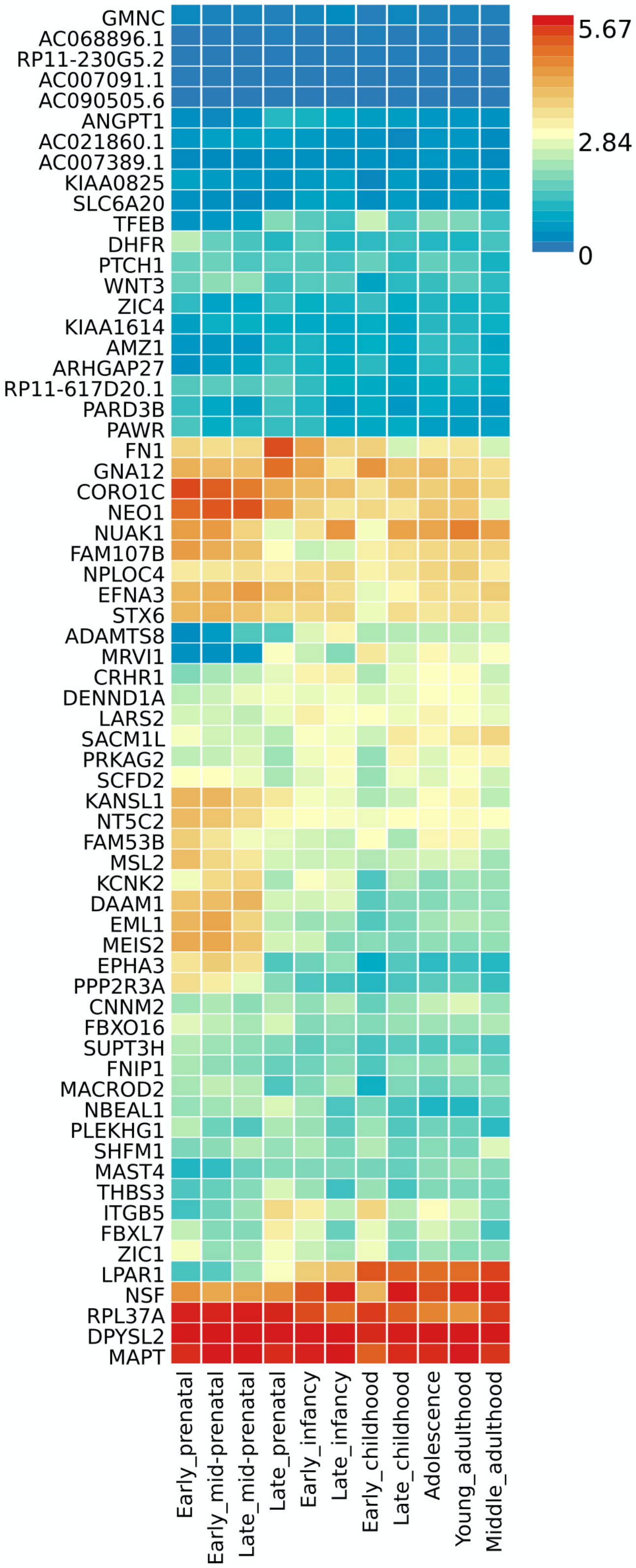

Supplementary Figure 8. (a) Differential expression (GTEx v8 gene expression data) of the closest genes to each lead GWAS variant across adult tissues. Tissues with significant (FDR $p \leq 0.05$) up- and down- regulation of genes within the closest gene set compared with other tissues are shown in red. **(b) Differential expression (Zhong et al 2018 single-cell RNA-seq expression data) of the closest genes to each lead GWAS variant across early to mid-gestation fetal brain cells sampled between gestation weeks 8-26**. Cells with significant (FDR $p \leq 0.05$) enrichment of gene expression of the closest gene set compared with other tissues are shown in red. *P*-values (uncorrected) derived from hypergeometric tests.

a

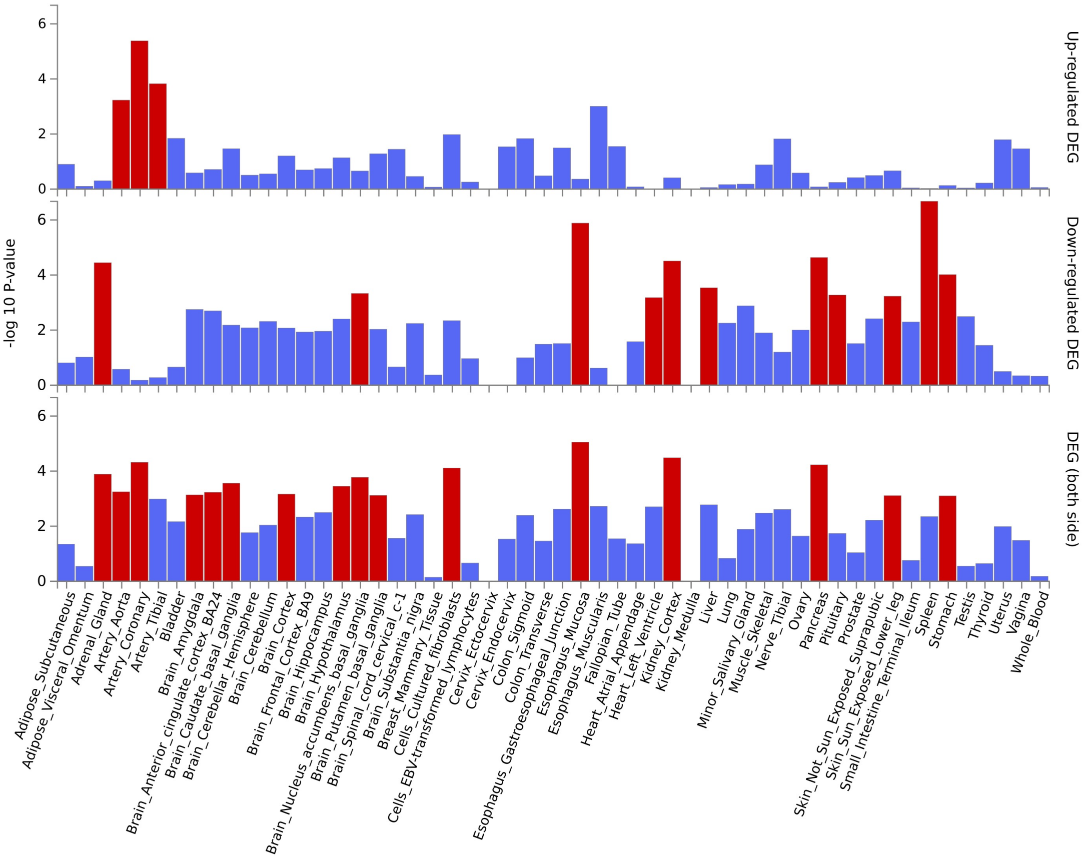

b

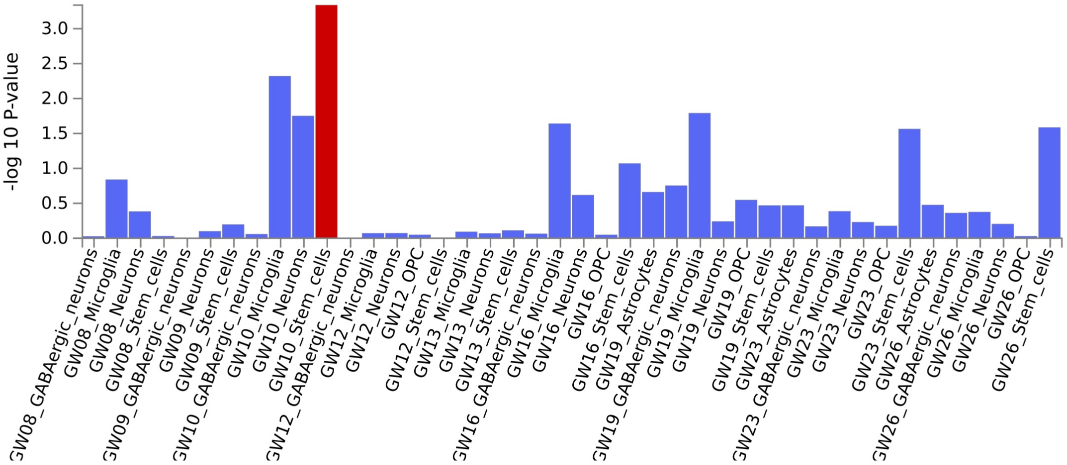

Supplementary Figure 9. Colocalization heatmap (PP4>0.5 shown) **of brain tissue *cis* eQTLs against significant brain sulcal associations.**

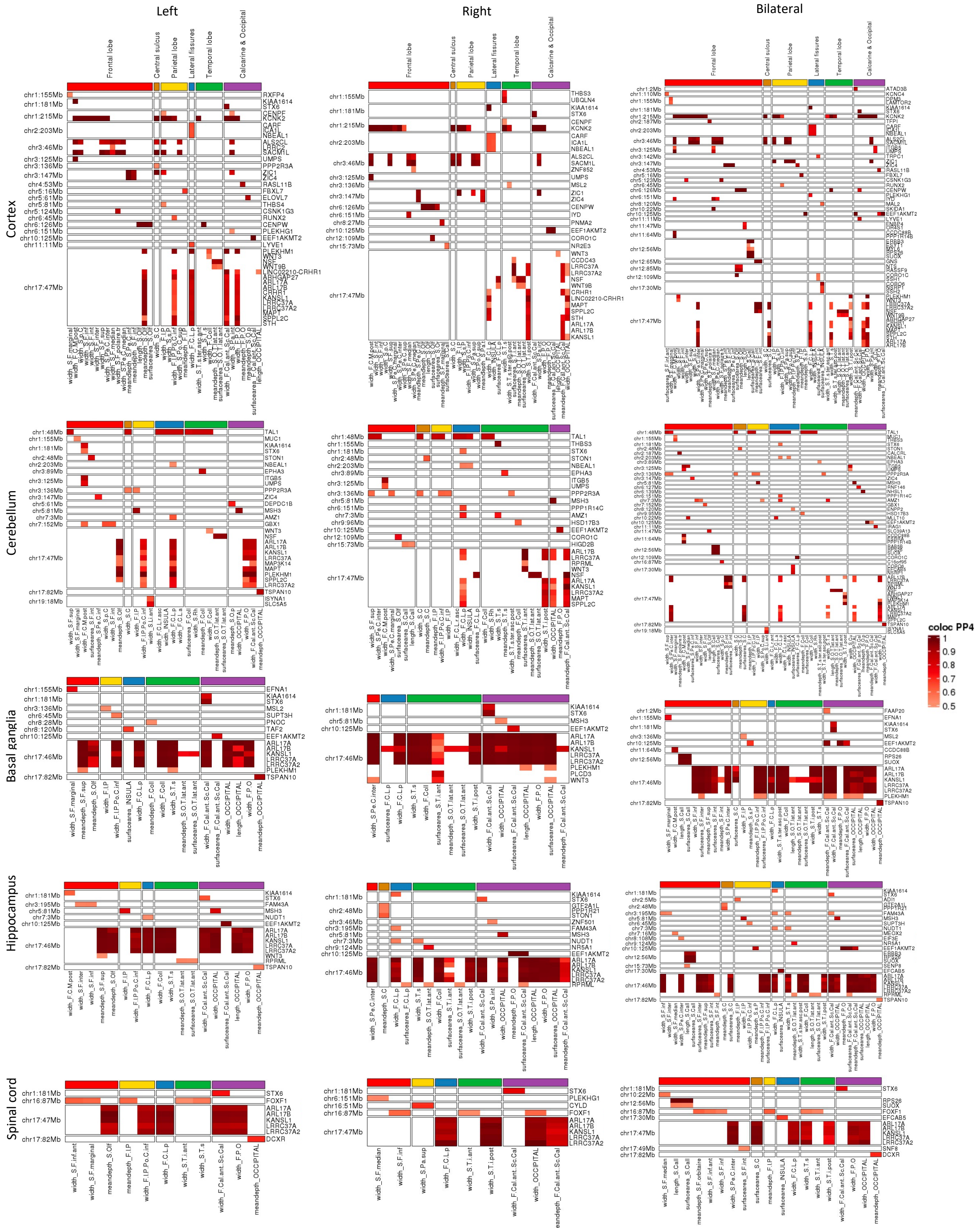

Summary of the reliability of the sulcal measurements

The reliability of sulcal measurements has been assessed previously[1] by estimating a) the dimensionless measure of absolute percent bias of the descriptor of a sulcus with respect to its average; and b) the ICC coefficient, i.e., the intra-class correlation coefficient, across four independent test-retest cohorts (HCP, QTIM, KKI, and OASIS). The bias should be close to zero for reliable metrics. The ICC provides an adequate relation of within-subject (σWS) and between-subject (σBS) variability[2-4]. In a test-retest framework, the σWS variability should be small compared to the σBS variability. Thus, ICC values below 0.4 are typically classified as having "poor" reproducibility, between 0.4 and 0.75 "fair to good," and higher values as "excellent" reproducibility[5]. A trade-off between test-retest data availability and requirements for the reproducibility analysis had to be made. An inter-scan interval < 90 days between test and retest scans was selected, ensuring that no anatomical changes occurred in participants' brains.

Empirical estimation of false discoveries

Here we motivate our choice of nominal test size α used in both the discovery phase, Bonferroni corrected $\alpha=5x10^{-8}$, as well as the replication phase $\alpha=0.05$. To do this, we generated a sample of test-statistics under the null of no association via a permutation test, achieved by permuting sample labels. This provides null data that preserves both the correlation structure between the phenotypes and the linkage disequilibrium (LD) between the genetic variants. For each permuted set, we performed GWAS and replication, followed by clumping, similar to our main analysis. The cardinality of the set of all possible permutations is beyond what is computationally feasible, rendering an exact permutation test intractable. To overcome this limitation, in a pragmatic sense, we computed a sample of test statistics under the null for a random subset of 100 permutations, allowing for an estimate of the empirical false positive rate beyond the nominal 5% significance level whilst keeping computation tractable. Results were then used to approximate the expected number of positives under the null.

In keeping with the previous large scale multi-phenotype brain imaging studies with a similar study design and thresholds[6,7], we found that empirical false discovery rates are well maintained at <5% in the combined discovery phase ($p<5x10^{-8}$) and replication phase ($p<0.05$, with concordant directions), with median false discovery rate of ~1.0% and 99-percentile false discovery rate of ~2.1%. As expected, using the more stringent multiple correction-adjusted discovery threshold of $2x10^{-10}$, the 99-percentile false discovery rate was ~0.54%.

Supplementary Figure 10. Empirical false discovery rates from permutation testing (n=100 times) at $p_{dis}<5x10^{-8}$ or $p_{dis}<2x10^{-10}$, with $p_{rep}<0.05$. Red and blue lines indicate the median and 99th percentiles respectively.

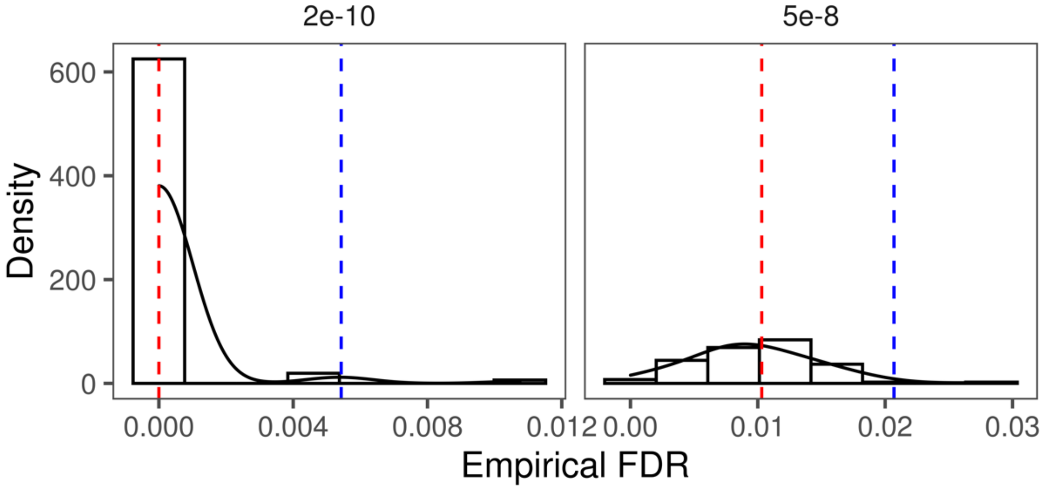
2e-10
5e-8
Density
600
400
200
0
0.000
0.004
0.008
0.012
0.00
0.01
0.02
0.03
Empirical FDR

Sensitivity analyses

Single X/Y/Z plane head scaling

We performed additional sensitivity analyses to investigate the potential effects of head scaling in the X, Y and Z planes as additional covariates in the genetic analyses of the lead associations. Z-scores were well-aligned with no systematic global deviations between sensitivity analyses (**Supplementary Figure 11a**). The strength of genetic associations (Z-scores) between additional X/Y/Z adjustments and adjusting for intracranial volume (ICV) only are high comparable, suggesting ICV adjustment adequately controls for X/Y/Z scaling of the brain implicitly.

Cortical thickness and surface area

We performed additional sensitivity analyses by adjusting for both cortical thickness and surface area, along with ICV. Most of the associations were well aligned with no systematic shifts in Z-scores globally. A total of 5 of 723 lead associations (388 sided and 335 bilateral-averaged) became completely null (p>0.05) and 59 of the 723 lead associations did not meet the suggestive $p > 1\text{x}10^{-6}$ after sensitivity adjustments (**Supplementary Figure 11b**). Broadly, these results are consistent with sulcal measures being distinct phenotypes relative to cortical measures, therefore providing some additional insights beyond cortical measures alone. Partial overlap in genetic associations is expected, as there should exist some shared genetic drivers of both cortical tissue morphology and sulcal formation. However, the lack of systematic shift across the entire Z-score range suggests that the associations we observed with sulcal measures were not strictly secondary to consequences of effects on cortical thickness/surface area.

Supplementary Figure 11. Sensitivity analysis of lead associations. (a) Associations adjusted for X/Y/Z scaling. (b) Associations adjusted for cortical thickness and surface area (y-axis). Z-score derived from regression GWAS.

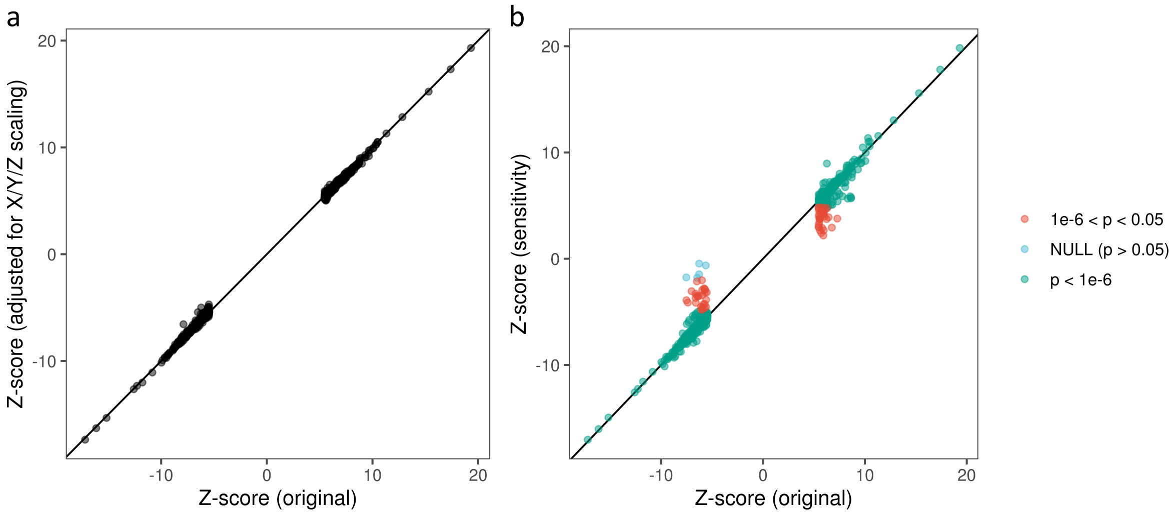
a
b
Z-score (adjusted for X/Y/Z scaling)
Z-score (original)
Z-score (sensitivity)
Z-score (original)
1e-6 < p < 0.05
NULL (p > 0.05)
p < 1e-6

Missense variant in *SLC6A20* transporter highlights role of glycine and proline pathways in brain sulcal width modulation

We discovered the missense variant rs17279437, causing Thr199Met in *SLC6A20*, to be associated, either as the lead variant or strong proxies ($r^2>0.8$), with widespread reduced sulcal widths (**Supplementary Data 5 and Supplementary Figure 12**). SLC6A20 is an amino-acid (especially proline/glycine) co-transporter expressed in the kidneys, intestines and brain[8]. This transporter has recently identified to play a role in brain glycine homeostasis and NMDA-type glutamate receptor (NMDAR) function[9]. Thr199Met SLC6A20 has a greatly reduced transport capacity compared with the wild-type protein[10] and has been implicated in iminoglycinuria and hyperglycinuria (abnormally proline, hydroxyproline and glycine levels in the urine)[11], consistent with *SLC6A20* expression and reduced reabsorption in renal tubules. Metabolomic QTL (mQTL) studies have also shown *SLC6A20* Thr199Met associations with urinary levels and ratios involving glycine derivatives[12,13] and with blood levels of pyroglutamate[14]. Within the nervous system, *SLC6A20* Thr199Met has been recently associated with increased levels of betaine (trimethylglycine)[15] and L-proline[16] in cerebrospinal fluid. In addition to widespread reduced sulcal widths, the Thr199Met variant is also associated with reduced macular thickness[17], reduced retinal nerve fibre layer and ganglion cell inner plexiform layer thicknesses[18] and increased risk of macular telangiectasia type 2[19] in the eye.

Together, these results suggest the role of SLC6A20 and glycine/proline related pathways in conditions related to retinal thicknesses or reduced sulcal widths, such as macular telangiectasia (type 2) or neurodegenerative and psychiatric conditions, in addition to renal conditions such as iminoglycinuria/hyperglycinuria.

Supplementary Figure 12. Association (Z-scores) of rs17279437 (*SLC6A20*) on sulcal widths in the brain.

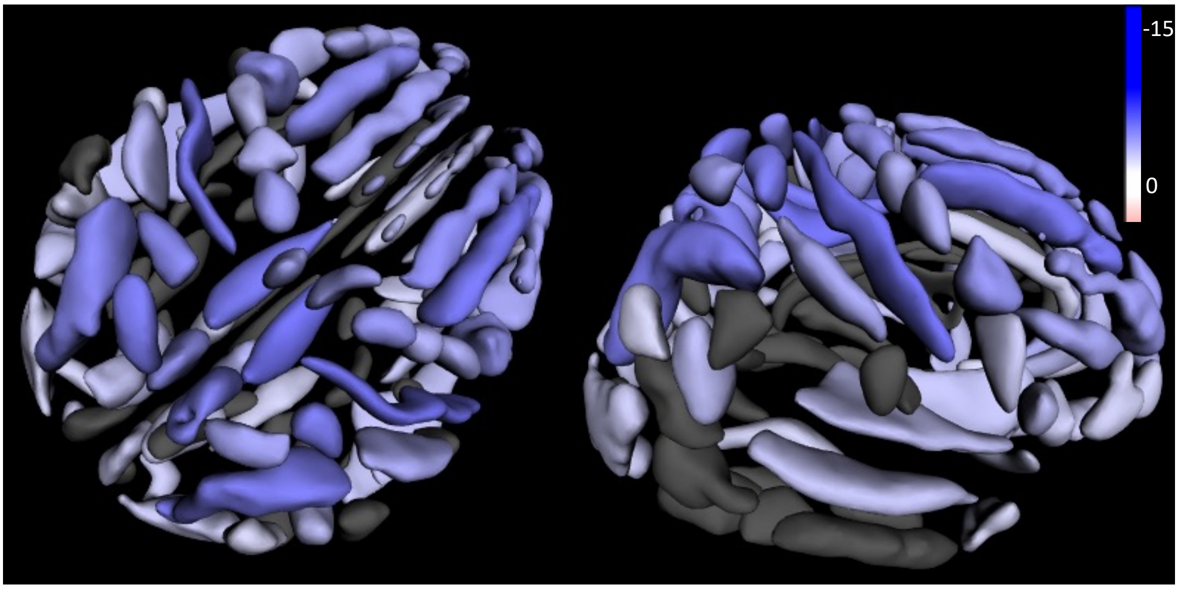

Supplementary Table 1. Summary of brain imaging related studies in GWAS Catalog

PMID	First Author	Year	Title
**33723403**	Sha Z	2021	The genetic architecture of structural left-right asymmetry of the human brain.
**33293549**	Sargurupremraj M	2020	Cerebral small vessel disease genomics and its implications across the lifespan.
**32963231**	Hofer E	2020	Genetic correlations and genome-wide associations of cortical structure in general population samples of 22,824 adults.
**32665545**	van der Meer D	2020	Understanding the genetic determinants of the brain with MOSTest.
**32358547**	Persyn E	2020	Genome-wide association study of MRI markers of cerebral small vessel disease in 42,310 participants.
**32198502**	Shin J	2020	Global and Regional Development of the Human Cerebral Cortex: Molecular Architecture and Occupational Aptitudes.
**32193296**	Grasby KL	2020	The genetic architecture of the human cerebral cortex.
**31676860**	Zhao B	2019	Genome-wide association analysis of 19,629 individuals identifies variants influencing regional brain volumes and refines their genetic co-architecture with cognitive and mental health traits.
**31666681**	Zhao B	2019	Large-scale GWAS reveals genetic architecture of brain white matter microstructure and genetic overlap with cognitive and mental health traits (n=17,706).
**31636452**	Satizabal CL	2019	Genetic architecture of subcortical brain structures in 38,851 individuals.
**31396565**	van der Lee SJ	2019	A genome-wide association study identifies genetic loci associated with specific lobar brain volumes.
**30818988**	Klein M	2019	Genetic Markers of ADHD-Related Variations in Intracranial Volume.
**30649180**	Luo Q	2019	Association of a Schizophrenia-Risk Nonsynonymous Variant With Putamen Volume in Adolescents: A Voxelwise and Genome-Wide Association Study.
**30305740**	Elliott LT	2018	Genome-wide association studies of brain imaging phenotypes in UK Biobank.
**30279459**	van der Meer D	2018	Brain scans from 21,297 individuals reveal the genetic architecture of hippocampal subfield volumes.
**30258056**	Vojinovic D	2018	Genome-wide association study of 23,500 individuals identifies 7 loci associated with brain ventricular volume.
**28924203**	Ren HY	2017	The common variants implicated in microstructural abnormality of first episode and drug-naive patients with schizophrenia.
**25607358**	Hibar DP	2015	Common genetic variants influence human subcortical brain structures.
**22504421**	Bis JC	2012	Common variants at 12q14 and 12q24 are associated with hippocampal volume
**22504418**	Ikram MA	2012	Common variants at 6q22 and 17q21 are associated with intracranial volume.

Multi-trati colocalization (HyPrColoc) sensitivity analysis

To assess sensitivity of our result using the default settings, we repeated our analyses across a range of parameter specifications (i.e., we performed a 3-dimensional grid search with p_c=[0.02, 0.01, 0.005], P_R=[0.6, 0.7, 0.8], P_A=[0.6, 0.7, 0.8]). Of the 56 traits assessed (cortical eQTL and all significant associations in the KCNK2 region) and across the range of parameter settings, the traits regularly formed a single cluster of jointly colocalized traits driven by the rs1452628 variant. Occasionally, for the smallest choice of prior and largest values of threshold parameters, the "bilateral Primary intermediate ramus of the intraparietal sulcus (F.I.P.r.int.1) width" measure was removed from the cluster.

Supplementary Table 2. Summary of neuropsychiatric and cognitive phenotypes tested for genetic correlation

Neuro trait	First author	Year	PMID	Sample size
**Alzheimer's disease**	Kunkle BW	2019	30820047	63,926
**Epilepsy**	Abou-Khalil B	2018	30531953	44,889
**ADHD**	Demontis D	2019	30478444	55,374
**Cognitive performance**	Lee JJ	2018	30038396	257,828
**Bipolar disorder**	Ruderfer DM	2018	29906448	74,194
**Major depressive disorder**	Wray NR	2018	29700475	173,005
**Autism spectrum disorder**	Anney RJL	2017	28540026	15,954
**Anorexia**	Duncan LE	2017	28494655	14,477
**Chronotype**	Jones SE	2016	27494321	127,898
**Generalized Anxiety disorder**	Otowa T	2016	26754954	17,310
**Parkinson's disease**	Nalls MA	2014	25064009	108,990
**Schizophrenia**	Ripke S	2014	25056061	77,096

Supplementary Table 3. Summary of prior peer-reviewed applications of BrainVISA to investigate brain sulcal morphology in human health and disease.

Phenotype	Main findings	Reference	PMID
**Very preterm birth (VPTB)**	Secondary sulci depths were significantly reduced in VPTB vs. controls	Giménez et al., Neurology, 2006	17130415
**Cerebral autosomal-dominant arteriopathy with subcortical infarcts and leukoencephalopathy (CADASIL)**	Brainvisa can be applied reliably to detect brain parenchymal fraction in CADASIL patients	O'Sullivan et al., Neuroimage, 2008	18722537
**Epilepsy**	MRI-negative epilepsy patients showed subclinical abnormal gyration patterns in the epileptogenic zone	Régis et al., Neurosurgery, 2011	21346658
**Parkinson's disease (PD)**	PD patients showed significant decreases of cortical folding in the left frontal and right collateral sulci vs. controls	Braga Pereira, Human Brain Mapping, 2012	21898679
**Alzheimer's disease (AD)**	Fold opening of the parietal-occipital fissure and intraparietal sulcus increased in early AD and AD vs. healthy controls	Reiner et al., J Alzheimers Dis., 2012	22297645
**Autism spectrum disorder (ASD)**	ASD patients showed greater surface area and length in the insula and intraparietal sulcus vs. controls	Shokouhi et al., Autism Res., 2012	22674695
**Brain development during adolescence**	The human cerebral cortex flattens during adolescence	Alemán-Gómez et al., J. Neuroscience, 2013	24048830
**Gilles de la Tourette syndrome**	Patients with Gilles de la Tourette syndrome exhibited lower sulcal depth in the pre- and post-central sulci and the superior, inferior and internal frontal sulci	Muellner et al., Movement Disorders, 2015	25820811
**Alzheimer's disease (AD)**	Left hemispheric cortical sulcal features can be used as biomarkers in AD, with an AUC of 0.89 in distinguishing AD patients from cognitively normal individuals	Plocharski et al., Comput Methods Programs Biomed., 2016	27393798
**Cerebral autosomal-dominant arteriopathy with subcortical infarcts and leukoencephalopathy (CADASIL)**	Focal macroscopic cortical lesions were confirmed in 11% of CADASIL patients but 0% of controls	Lyoubi-Idrissi et al., Stroke, 2017	28348068
**Congenital blindness**	Central sulcus morphology profiles differ in congenitally blind vs. sighted individuals	James et al., Annu Int Conf IEEE Eng Med Biol Soc., 2017	29060531
**Alzheimer's disease (AD)**	Sulcal width can differentiate posterior cortical atrophy from typical AD	Fumagalli GG et al., Neuroimage Clinical, 2020	33045537
**Isolated growth hormone deficiency (IGHD)**	Bilateral average width of the central sulci was higher, and depth was lower, in children with IGHD vs. idiopathic short stature	Zhang et al., Dev Neurobiol, 2021	33277816

Biogen Biobank Team contributors

Steering team: Ellen Tsai, Christopher D. Whelan, Paola Bronson, David Sexton, Sally John, Heiko Runz.
Data management team: Eric Marshall, Mehool Patel, Saranya Duraisamy, Timothy Swan.
Extended scientific team: Dennis Baird, Chia-Yen Chen, Susan Eaton, Jake Gagnon, Feng Gao, Cynthia Gubbels, Yunfeng Huang, Varant Kupelian, Kejie Li, Dawei Liu, Stephanie Loomis, Helen McLaughlin, Adele Mitchell, Benjamin Sun.

Supplementary References

1 Pizzagalli, F. *et al.* The reliability and heritability of cortical folds and their genetic correlations across hemispheres. *Commun Biol* **3**, 510, doi:10.1038/s42003-020-01163-1 (2020).

2 Andreotti, J. *et al.* Validation of network communicability metrics for the analysis of brain structural networks. *PLoS One* **9**, e115503, doi:10.1371/journal.pone.0115503 (2014).

3 Lachin, J. M. The role of measurement reliability in clinical trials. *Clin Trials* **1**, 553-566, doi:10.1191/1740774504cn057oa (2004).

4 Shrout, P. E. & Fleiss, J. L. Intraclass correlations: uses in assessing rater reliability. *Psychol Bull* **86**, 420-428, doi:10.1037//0033-2909.86.2.420 (1979).

5 Landis, J. R. & Koch, G. G. The measurement of observer agreement for categorical data. *Biometrics* **33**, 159-174 (1977).

6 Elliott, L. T. *et al.* Genome-wide association studies of brain imaging phenotypes in UK Biobank. *Nature* **562**, 210-216, doi:10.1038/s41586-018-0571-7 (2018).

7 Smith, S. M. *et al.* An expanded set of genome-wide association studies of brain imaging phenotypes in UK Biobank. *Nat Neurosci* **24**, 737-745, doi:10.1038/s41593-021-00826-4 (2021).

8 Broer, S. The SLC6 orphans are forming a family of amino acid transporters. *Neurochem Int* **48**, 559-567, doi:10.1016/j.neuint.2005.11.021 (2006).

9 Bae, M. *et al.* SLC6A20 transporter: a novel regulator of brain glycine homeostasis and NMDAR function. *EMBO Mol Med* **13**, e12632, doi:10.15252/emmm.202012632 (2021).

10 Broer, S. *et al.* Iminoglycinuria and hyperglycinuria are discrete human phenotypes resulting from complex mutations in proline and glycine transporters. *J Clin Invest* **118**, 3881-3892, doi:10.1172/JCI36625 (2008).

11 Yahyaoui, R. & Perez-Frias, J. Amino Acid Transport Defects in Human Inherited Metabolic Disorders. *Int J Mol Sci* **21**, doi:10.3390/ijms21010119 (2019).

12 Raffler, J. *et al.* Genome-Wide Association Study with Targeted and Non-targeted NMR Metabolomics Identifies 15 Novel Loci of Urinary Human Metabolic Individuality. *PLoS Genet* **11**, e1005487, doi:10.1371/journal.pgen.1005487 (2015).

13 Suhre, K. *et al.* A genome-wide association study of metabolic traits in human urine. *Nat Genet* **43**, 565-569, doi:10.1038/ng.837 (2011).

14 Shin, S. Y. *et al.* An atlas of genetic influences on human blood metabolites. *Nat Genet* **46**, 543-550, doi:10.1038/ng.2982 (2014).

15 Panyard, D. J. *et al.* Cerebrospinal fluid metabolomics identifies 19 brain-related phenotype associations. *Commun Biol* **4**, 63, doi:10.1038/s42003-020-01583-z (2021).

16 Luykx, J. J. *et al.* Genome-wide association study of NMDA receptor coagonists in human cerebrospinal fluid and plasma. *Mol Psychiatry* **20**, 1557-1564, doi:10.1038/mp.2014.190 (2015).

17 Gao, X. R., Huang, H. & Kim, H. Genome-wide association analyses identify 139 loci associated with macular thickness in the UK Biobank cohort. *Hum Mol Genet* **28**, 1162-1172, doi:10.1093/hmg/ddy422 (2019).

18 Currant, H. *et al.* Genetic variation affects morphological retinal phenotypes extracted from UK Biobank optical coherence tomography images. *PLoS Genet* **17**, e1009497, doi:10.1371/journal.pgen.1009497 (2021).

19 Bonelli, R. *et al.* Identification of genetic factors influencing metabolic dysregulation and retinal support for MacTel, a retinal disorder. *Commun Biol* **4**, 274, doi:10.1038/s42003-021-01788-w (2021).
