# Supplementary material for: Genetic map of regional sulcal morphology in the human brain from UK biobank data
Source: Nat Commun. 2022 Oct 14;13:6071. doi: 10.1038/s41467-022-33829-1 (PMC9568560; doi:10.1038/s41467-022-33829-1)
Supplement: Supplementary file 3 — Description of Additional Supplementary Files [file 41467_2022_33829_MOESM3_ESM.pdf]

Description of Additional Supplementary Files

Supplementary Data-12:

Supplementary Data 1 • SD1. Brain folding map nomenclature. List of the 62 sulcal regions under investigation, including abbreviated name and full name.

Supplementary Data 2 • SD2. List of UK Biobank measurements. List of imaging-derived phenotypes in the UK Biobank used in t-SNE clustering analysis.

Supplementary Data 3 • SD3. GWAS associations. All genome-wide significant associations, replicated at $p<0.05$, across specific brain regions.

Supplementary Data 4 • SD4. Brain folding map nomenclature. All genome-wide significant associations, replicated at $p<0.05$, averaged across hemispheres.

Supplementary Data 5 • SD5. GWAS associations summary. Summary of number of significant associations per sulcal shape parameter/hemisphere.

Supplementary Data 6 • SD6. Multi-trait colocalization results. Results of multi-trait colocalization analysis, for loci associated with >1 sulcal measure.

Supplementary Data 7 • SD7. Overlapping brain imaging GWAS loci. List of genetic associations from the present study that overlap with previously reported brain imaging GWAS associations.

Supplementary Data 8 • SD8. Coding proxy associations. GWAS associations in linkage disequilibrium (LD; $r^2>0.8$) with coding variants or splice site proxies.

Supplementary Data 9 • SD9. Burden analysis results. List of rare variant burden association results at $p<2.7x10^{-6}$.

Supplementary Data 10 • SD10. Expression quantitative trait loci (eQTL) colocalization results. Results of eQTL colocalization analysis at PP4>0.5.

Supplementary Data 11 • SD11. GWAS Catalog overlap. List of associations overlapping with genetic associations reported in the NHGRI-EBI GWAS Catalog (ebi.ac.uk/gwas/).

Supplementary Data 12 • SD12. Neurocognitive correlations. Genetic correlations between measures of sulcal morphometry and neurocognitive phenotypes, at $p<0.05$.
