# Supplementary material for: Genetic map of regional sulcal morphology in the human brain from UK biobank data
Source: Nat Commun. 2022 Oct 14;13:6071. doi: 10.1038/s41467-022-33829-1 (PMC9568560; doi:10.1038/s41467-022-33829-1)
Supplement: Supplementary file 5 — Reporting Summary [file 41467_2022_33829_MOESM5_ESM.pdf]

nature portfolio

Corresponding author(s): Benjamin Sun, Chris Whelan, Paul Thompson, Neda Jahanshad

Last updated by author(s): Sep 19, 2022

Reporting Summary

Nature Portfolio wishes to improve the reproducibility of the work that we publish. This form provides structure for consistency and transparency in reporting. For further information on Nature Portfolio policies, see our Editorial Policies and the Editorial Policy Checklist.

Statistics

For all statistical analyses, confirm that the following items are present in the figure legend, table legend, main text, or Methods section.

n/a	Confirmed	
☐	☒	The exact sample size (n) for each experimental group/condition, given as a discrete number and unit of measurement
☐	☒	A statement on whether measurements were taken from distinct samples or whether the same sample was measured repeatedly
☐	☒	The statistical test(s) used AND whether they are one- or two-sided<br>*Only common tests should be described solely by name; describe more complex techniques in the Methods section.*
☐	☒	A description of all covariates tested
☐	☒	A description of any assumptions or corrections, such as tests of normality and adjustment for multiple comparisons
☐	☒	A full description of the statistical parameters including central tendency (e.g. means) or other basic estimates (e.g. regression coefficient) AND variation (e.g. standard deviation) or associated estimates of uncertainty (e.g. confidence intervals)
☐	☒	For null hypothesis testing, the test statistic (e.g. F, t, r) with confidence intervals, effect sizes, degrees of freedom and P value noted<br>*Give P values as exact values whenever suitable.*
☐	☒	For Bayesian analysis, information on the choice of priors and Markov chain Monte Carlo settings
☒	☐	For hierarchical and complex designs, identification of the appropriate level for tests and full reporting of outcomes
☐	☒	Estimates of effect sizes (e.g. Cohen's d, Pearson's r), indicating how they were calculated

Our web collection on statistics for biologists contains articles on many of the points above.

Software and code

Policy information about availability of computer code

Data collection	Data collection and processing detailed in Methods and in reference 67. No other software used for data collection.
Data analysis	R v3.6.1 including packages: TissueEnrich v1.6.0, GenomicRanges v1.38.0, ComplexHeatmap v2.11.1, coloc v5.1.0, hyprcoloc v1.0.<br>REGENIE v2.0.1<br>PLINK v1.9<br>LDSC v1.0.1<br>FreeSurfer v7.1.1<br>BrainVISA v4<br>Details of specific software and references can be found within text in the relevant Methods.

For manuscripts utilizing custom algorithms or software that are central to the research but not yet described in published literature, software must be made available to editors and reviewers. We strongly encourage code deposition in a community repository (e.g. GitHub). See the Nature Portfolio guidelines for submitting code & software for further information.

Data

Policy information about availability of data

All manuscripts must include a data availability statement. This statement should provide the following information, where applicable:

- Accession codes, unique identifiers, or web links for publicly available datasets
- A description of any restrictions on data availability
- For clinical datasets or third party data, please ensure that the statement adheres to our policy

The online browser for visualisation of results and links to summary association data and is available at https://enigma-brain.org/sulci-browser. Human Protein Atlas

(https://www.proteinatlas.org/). BrainSpan (https://www.brainspan.org/). MetaBrain (https://www.metabrain.nl/). GTEx (https://gtexportal.org/home/).

Field-specific reporting

Please select the one below that is the best fit for your research. If you are not sure, read the appropriate sections before making your selection.

☒ Life sciences ☐ Behavioural & social sciences ☐ Ecological, evolutionary & environmental sciences

For a reference copy of the document with all sections, see nature.com/documents/nr-reporting-summary-flat.pdf

Life sciences study design

All studies must disclose on these points even when the disclosure is negative.

Sample size

Methods, "Samples and participants" and "Discovery and replication cohorts". This is a discovery study across multiple phenotypes and thus we did not perform specific power calculations to detect pre-specified effect sizes. Sample sizes were based on all available data in UK Biobank with MRI imaging.

Data exclusions

Exclusions during QC process (phenotypic and genetic) detailed in page 20-24 in Methods.

Brain folding imaging phenotypes
The UK Biobank began collecting brain MRI scans in 2014 with the goal of scanning 100,000 individuals. The protocol includes isotropic 3D T1-weighted (T1w) MP-RAGE images (voxel size 1 mm3; field-of-view: 208 x 256 x 256) that have undergone bias-field correction in the scanner. Full acquisition details can be found in67. T1w images were processed using FreeSurfer (v7.1.1) and quality controlled using protocols developed by the Enhancing Neuro Imaging Genetics for Meta-Analysis (ENIGMA) consortium (http://enigma.ini.usc.edu/). BrainVISA (http://brainvisa.info) was implemented for sulcal classification and labelling65,68. Prior applications of BrainVISA to study human brain development and disease are summarised in Supplementary Table 13. Morphologist 2015, an image-processing pipeline included in BrainVISA, was used to measure sulcal shape descriptors. To improve sulcal extraction and build on current protocols used to analyse thousands of brain scans, quality controlled FreeSurfer outputs (orig.mgz, ribbon.mgz and talairach.auto) were directly imported into the pipeline to avoid re-computing intensities inhomogeneities correction and grey/white matter classification. Sulci were then automatically labelled according to a predefined anatomical nomenclature68,69. This protocol is part of the ENIGMA-SULCI working group; a Docker and a Singularity container have been created to facilitate the processing on computational clusters (https://hub.docker.com/repository/docker/fpizzaga/sulci). We retained length, width, depth, and surface area for all 121 sulcal measurements derived from this protocol for a total of 484 phenotypes (https://surfer.nmr.mgh.harvard.edu/) and). BrainVISA (http://brainvisa.info) was implemented for sulcal classification and labelling68. Prior applications of the BrainVISA tool to study health and disease are summarised in the Morphologist 2015, an image-processing pipeline included in BrainVISA, was used to measure sulcal shape descriptors. To improve sulcal extraction and build on current protocols used to analyse thousands of brain scans, quality controlled FreeSurfer outputs (orig.mgz, ribbon.mgz and talairach.auto) were directly imported into the pipeline to avoid re-computing intensities inhomogeneities correction and grey/white matter classification. Sulci were then automatically labelled according to a predefined anatomical nomenclature70. This protocol is part of the ENIGMA-SULCI working group; a Docker and a Singularity container have been created to facilitate the processing on computational clusters (https://hub.docker.com/repository/docker/fpizzaga/sulci). We retained length, width, depth, and surface area for all 121 sulcal measurements derived from this protocol for a total of 484 phenotypes.http://enigma.ini.usc.edu/).68,70

Phenotypes with missingness >75% were excluded from subsequent analysis, leaving 450 measurements (224 left and 225 right hemisphere measures) for analysis. Missingness occurs mainly with smaller sulci that are not identified in some individual MRIs. Prior to analysis, all imaging phenotypes were inverse-rank normalised to approximate a standard normal distribution and minimise effects of outliers.

UKB genetic QC
UKB genotyping and imputation (and QC) were performed as described previously66. WES data for UKB participants were generated at the Regeneron Genetics Center (RGC) as part of a collaboration between AbbVie, Alnylam Pharmaceuticals, AstraZeneca, Biogen, Bristol-Myers Squibb, Pfizer, Regeneron and Takeda with the UK Biobank73. WES data were processed using the RGC SBP pipeline as described in74,75. RGC generated a QC-passing “Goldilocks” set of genetic variants from a total of 454,803 sequenced UK Biobank participants for analysis. Additional QC were performed prior to association analyses as detailed below.

Additional QC and variant processing
In addition to checking for sex mismatch, sex chromosome aneuploidy, and heterozygosity checks, imputed genetic variants were filtered for INFO>0.8, MAF>0.01 (rarer variants around coding regions would be better captured by WES) globally across UKB and chromosome positions were lifted to hg38 build. WES variants were filtered for MAC>10 within the UKB subset with MRI measurements. Imputed and WES variants were combined by chromosome position (hg38) and alleles and in the case of overlaps, the WES variant was retained (as WES generally have higher quality calls compared to imputation). Variant annotation was performed using VEP76 with Ensembl canonical transcripts used where possible.

Replication

We employed a replication cohort (detailed in Methods - "Discovery and replication cohorts"). Replication of lead discovery associations for directionally concordant associations in the replication cohort at $p<0.05$. Replication samples are independent biological participant samples. replication results are detailed in Results.

Randomization

No experimental vs control group per se.

Blinding

No experimental vs control group per se. All data are anonymised and analysts were blind to sample statuses.

Reporting for specific materials, systems and methods

We require information from authors about some types of materials, experimental systems and methods used in many studies. Here, indicate whether each material, system or method listed is relevant to your study. If you are not sure if a list item applies to your research, read the appropriate section before selecting a response.

Materials & experimental systems

n/a	Involved in the study
☒	☐ Antibodies
☒	☐ Eukaryotic cell lines
☒	☐ Palaeontology and archaeology
☒	☐ Animals and other organisms
☐	☒ Human research participants
☒	☐ Clinical data
☒	☐ Dual use research of concern

Methods

n/a	Involved in the study
☒	☐ ChIP-seq
☒	☐ Flow cytometry
☐	☒ MRI-based neuroimaging

Human research participants

Policy information about studies involving human research participants

Population characteristics

Details can be found Extended Data Figure 2. UKB population characteristics available at https://biobank.ndph.ox.ac.uk/showcase/. Also described in Methods ("Samples and participants"). UKB has been described in the public domain.

Age (years, mean [IQR]) 55.4 [49.0-61] - discovery 54.6 [49.0-61.0] - replication
Gender (% female) 51.3 - discovery 53.0 - replication
BMI (kg/m^2, mean [IQR]) 26.5 [23.6-28.8] - discovery 26.6 [23.7-28.9] - replication
Smoking (current) % 6.3 - discovery 6.1 - replication

All have genotypic information available

Recruitment

UKB is a population cohort sampled from across UK sites. Detailed in Methods ("Samples and participants", page 18)

Ethics oversight

Analyses in this study were conducted under UK Biobank Approved Project numbers 26041 and 11559.

Note that full information on the approval of the study protocol must also be provided in the manuscript.

Magnetic resonance imaging

Experimental design

Design type

Resting

Design specifications

Full details available at https://biobank.ctsu.ox.ac.uk/crystal/crystal/docs/brain_mri.pdf. Additional processing detailed in Methods - "Brain folding imaging phenotypes", page 18

Behavioral performance measures

NA

Acquisition

Imaging type(s)

Full details available at https://biobank.ctsu.ox.ac.uk/crystal/crystal/docs/brain_mri.pdf.

Field strength

Full details available at https://biobank.ctsu.ox.ac.uk/crystal/crystal/docs/brain_mri.pdf.

Sequence & imaging parameters

Full details available at https://biobank.ctsu.ox.ac.uk/crystal/crystal/docs/brain_mri.pdf.

Area of acquisition

Whole brain

Diffusion MRI ☐ Used ☒ Not used

Preprocessing

Preprocessing software

T1- wighted images were processed using FreeSurfer (v7.1.1) (https://surfer.nmr.mgh.harvard.edu/) and quality controlled using protocols developed by the Enhancing Neuro Imaging Genetics for Meta-Analysis (ENIGMA) consortium (http://enigma.ini.usc.edu/). BrainVISA 4.5 (http://brainvisa.info) was implemented for sulcal classification and labelling. Morphologist 2015, an image-processing pipeline included in BrainVISA, was used to measure sulcal shape descriptors. To improve sulcal extraction and build on current protocols used to analyse thousands of brain scans, quality controlled

	FreeSurfer outputs (orig.mgz, ribbon.mgz and talairach.auto) were directly imported into the pipeline to avoid re-computing several steps, including intensities inhomogeneities correction and gray/white matter classification. Sulci were then automatically labelled according to a predefined anatomical nomenclature of sulcal labels for the left hemisphere and sulcal labels for the right hemisphere. This protocol is part of the ENIGMA-SULCI working group; a Docker and a Singularity container have been created to facilitate the processing on computational clusters (https://hub.docker.com/repository/docker/fpizzaga/sulci).
Normalization	Sulcal measures were extracted in the native space of each subject.
Normalization template	-
Noise and artifact removal	-
Volume censoring	-

Statistical modeling & inference

Model type and settings	-
Effect(s) tested	-

Specify type of analysis: ☐ Whole brain ☒ ROI-based ☐ Both

Anatomical location(s)	Automatic segmentation and labeling algorithms were used as implemented in BrainVISA (https://brainvisa.info/web/). The sulci labeling model proposed in the BrainVISA/Morphologist package, referred as the Statistical Probabilistic Anatomy Map (SPAM) model, based on a Bayesian approach.
Statistic type for inference (See Eklund et al. 2016)	-
Correction	-

Models & analysis

n/a	Involved in the study
☒	☐ Functional and/or effective connectivity
☒	☐ Graph analysis
☒	☐ Multivariate modeling or predictive analysis
